# Role of Individual Heat Transfer Mechanisms Within a Model Baking Oven Heated by Porous Volumetric Ceramic Burners

**DOI:** 10.3389/fchem.2020.511012

**Published:** 2020-10-15

**Authors:** Vojislav Jovicic, Ana Zbogar-Rasic, Benedikt Burjakow, Antonio Delgado

**Affiliations:** ^1^Institute of Fluid Mechanics, Friedrich-Alexander University Erlangen-Nuremberg, Erlangen, Germany; ^2^Erlangen Graduate School in Advanced Optical Technologies (SAOT), Erlangen, Germany

**Keywords:** porous media burner, thermal radiation, infrared baking, baking oven, combustion in porous inert media

## Abstract

The baking process demands a high amount of energy, but only one-third of the total energy supply to the baking oven is actually used for baking, while the rest is dissipated to the environment. This implies that the energy input to the baking process can be significantly reduced, e.g., by enabling a more efficient heat transfer to the product, compared to commercially available ovens. Application of highly radiative, gas-fired heat sources, with a wide power modulation range, such as porous volumetric ceramic burners (VCB), can lead to a reduction in both the baking time and the energy input to a baking oven. In order to optimize energy input to a wide variety of baking products, the role of individual mechanisms in heat transfer between a heat source and a baking product needs to be determined. In the scope of this work, the analysis of the heat transfer within a baking oven model, heated by porous VCBs, was conducted. Contribution of heat transfer mechanisms (heat conduction, convection, thermal radiation) to the total heat transfer was determined by the difference method, where two aluminum cubes of different surface characteristics were used as target objects. Further, the influence of water, commonly added to the baking chamber in form of steam or aerosol, on the heat transfer characteristics within the oven was investigated. Without water addition, the heat transfer between the porous VCBs and the test object occurred mainly through thermal radiation (~45%), followed by heat conduction and convection (~27.5% each). Compared to the reference, commercially available electrical deck baking oven, the share of thermal radiation in the model oven was increased (+ 10%), whereas the share of heat conduction was reduced (−20%). With water addition, the heat transfer to the test object through heat conduction, convection, and thermal radiation declined, as an additional heat transfer through condensation took place. Results of this research provide necessary understanding of the heat transfer mechanisms within the novel baking oven, heated by porous VCBs. They are the base for optimization of the heat transfer from the VCBs to different baking goods, through changing the VCB's operating parameters.

## Introduction

The annual energy use of the food industry in Germany is ~58 TWh, out of which some 6 TWh is used for pastry production (Blesl and Kessler, 2017). The most energy-intensive step within the pastry production is the baking, with the average energy demand of 7 MJ per kg of bread (Fellows, 1996). An energy flow analysis of an average industrial bakery shows that over 50% of the energy supply is directed to baking ovens, but only one-third of it is used for baking, while the rest is dissipated to the environment (Schulz, 2014). Therefore, the energy-saving potential of the baking industry is correspondingly high, whereas the energy efficiency of baking ovens is the key factor for energy management within this food production branch.

The efficiency of the baking process can be increased by introducing novel baking concepts, which are able to provide better regulation of the main process parameters and a more efficient heat transfer to the product, compared to conventional baking ovens. As a result, total baking time, energy input (i.e., fuel consumption), and the resulting emissions of pollutants and greenhouse gases can be significantly decreased. One innovative approach could be the integration of a porous volumetric ceramic burner (VCB) technology in a baking oven, as was demonstrated elsewhere (Takacs et al., 2017; Jovicic and Delgado, 2018). Besides an extremely wide regulation range in terms of thermal power (1:20 or more) and, consequently, in terms of temperature, the radiation-to-convection heat transfer between this burner type and a heated object is high, compared to state-of-the-art ovens, with a positive effect on the baking process, e.g., preserved product quality at reduced baking time.

### Porous Volumetric Ceramic Burner (VCB)

**Figure 1:**
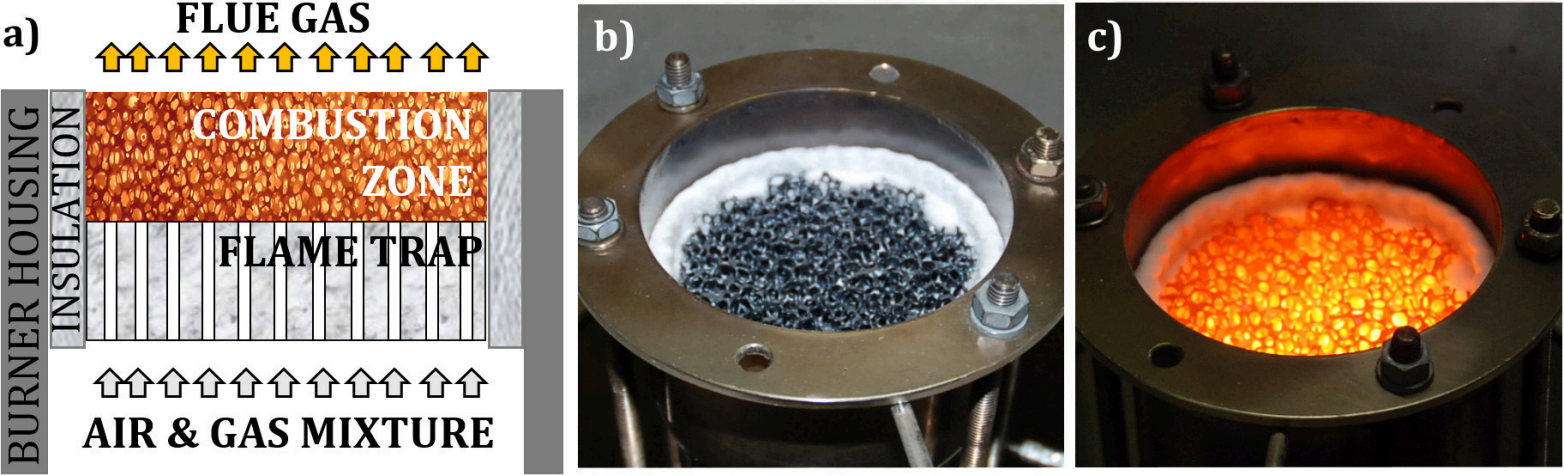
Porous VCB: **(a)** operating principle, **(b)** SiSiC—porous matrix of the VCB, and **(c)** VCB in operation.

Porous volumetric ceramic burner (VCB) is a combustion technology, where a gaseous, premixed air/fuel mixture combusts within cavities of a porous, inert material. Catapan et al. (2011) noted three flame stabilization mechanisms in porous burners: (1) thermal stabilization in a single ceramic foam, leading to plane, surface flames (Hsu et al., 1993), (2) quenching stabilization in a two-layered porous structure of different porosities, leading to a plane, submerged flame (Trimis and Durst, 1996), and (3) fluid dynamic stabilization, based on a non-uniform velocity profile and resulting in conical, jet-like flames (Francisco et al., 2010). Flame stabilization in a two-layer structure, which provides a simple, compact construction and applicability under a wide range of operating conditions, is applied in the scope of this work. Figure 1a shows two-layer porous VCB, consisting of a flame trap (zone with small pores, with the modified Pe number Pe <65) and a combustion zone (zone with big pores and Pe >65). Besides the prevention of the flame flashback through the flame quenching, the flame trap provides preheating of the incoming air–fuel mixture. Porous VCB is mainly produced from porous ceramic materials, e.g., SiSiC and Al_2_O_3_ (Pickenäcker et al., 1999; Mach et al., 2006), which can withstand high thermal stresses (during the ignition phase) and high temperatures (in operation). Figures 1b,c show a porous VCB, made of SiSiC, not in operation and in operation, respectively.

Porous VCB technology offers a number of advantages in comparison to a conventional free flame burner: stable operation over a wide range of power loads and air ratios, resulting in a high-power modulation range (up to 1:25), compact design due to high power density (up to 3 MW/m^2^), and preserved operation quality independent of burner orientation (Trimis et al., 2002, 2005; Jovicic et al., 2014; Raab et al., 2015). Due to the presence of a porous, ceramic material in the combustion zone, the effective heat transfer is much higher compared to free flame combustion, leading to a highly homogeneous temperature field, intensive heat coupling, and low pollutant emissions (CO, NO_x_) (Trimis et al., 2002, 2005), far below the “Blue Angel” norms. Due to the high temperature of a porous combustion zone (in the range of 1,000–1,500°C), porous VCB has a significantly higher thermal radiation output in the near-infrared range (NIR), especially in the wavelengths between 1,300 and 1,700 nm (Jovicic et al., 2014).

Porous VCB technology was previously successfully applied in different, mainly high-temperature applications, e.g., in glass or steel industry (Trimis et al., 2002; Mach et al., 2007; Altendorfner et al., 2008; Jovicic et al., 2010; Beier et al., 2011). Its application within the food industry is still not well-established, even though it could provide economic and environmental benefits to the production process through its unique thermal radiation and operating characteristics. Implementation of porous VCBs, as a heat source for a novel baking oven model, enables a significantly wider oven operation range and thus enables the implementation of a wide variety of baking programs.

### Infrared Baking

Heat transfer in baking ovens was analyzed in different studies (Standing, 1974; Krist-Spit and Sluimer, 1987; Baik et al., 1999). Among other parameters, it depends on the oven type, material, geometry, and heat source type (Baik et al., 1999; Ploteau et al., 2015). According to MIWE (2011), in a conventional multi-deck baking oven, the heat is transferred to the target object through thermal radiation (30%), conduction (40%), convection (15%), and condensation (15%). Various studies (Wade, 1987; Skjöldebrand and Andersson, 1989; Skjöldebrand, 2002; Olsson et al., 2005; Speer and John, 2006; Hermann et al., 2012) demonstrated a positive influence of thermal radiation on the baking process, e.g., overall reduction of the heat input, with an improved process regulation. According to Krishnamurthy et al. (2008), the baking process can profit from near-infrared radiation, due to its high penetration depth, thus producing the effect of “inside out” baking (Wade, 1987). It was shown that the use of thermal radiation decreased the baking time (Wade, 1987; Skjöldebrand and Andersson, 1989), e.g., for 10-cm flat pastry pieces, the reduction in baking time was 63%. In case of the STIR oven technology (Speer and John, 2006; Hermann et al., 2012), where the source of thermal radiation is the oven coating, the baking time was reduced to 20–50%, depending on the product. Regarding the product quality, the desired crust color can be reached faster, which was demonstrated on baking of the prebaked baguettes (Olsson et al., 2005) and white bread (Skjöldebrand and Andersson, 1989). The produced bread had a thinner crust and a softer crumb, in comparison to a bread baked in a conventional oven (Skjöldebrand and Andersson, 1989). Unsatisfactory results obtained in infrared baking ovens are less numerous than the positive ones. For example, Keskin et al. (2004) and Sumnu et al. (2005) claimed that infrared heating should not be used as a single heat source in the baking oven, as it results in a thick crust and a very low-strength crumb.

### Addition of Water During Baking

In order to reach a high-quality baking product, especially in terms of crust quality, rapid addition of water spray, fog, or steam to a baking chamber is desired at the beginning of the baking process (Meuser, 2016). Additionally, the heat transfer to baked goods depends on the humidity of a baking oven atmosphere, which is regulated by the addition of water.

According to Schünemann and Treu (2002), when steam enters the oven, which is heated above 150°C (at the beginning of the baking process), it condenses on surfaces of baked goods, as they are the coldest surfaces within the oven, having a temperature of ~30°C, i.e., well-below the dew point of water at atmospheric conditions. During this process, the enthalpy of condensation (equal to the latent heat *h*_*fg*__,H20_ = 2256.7 kJ/kg at 100°C and 1 bar) (Smith, 2003) will be transferred to the products' surface. In this way, high heat transfer rates are achieved with small temperature differences (Incropera and De Witt, 1996). Due to nearly atmospheric pressure within the oven, the condensation occurs at ~100°C. As soon as the surface temperature of the product exceeds the dew point temperature, the condensation of the excess water will stop.

Due to the released latent heat, the proteins in the dough immediately clot and the starch gelatinizes. This is the prerequisite for a brown and crunchy crust. Condensation promotes partial dextration of starch, which makes the pastry surface shiny (Meuser, 2016).

The amount and the duration of water added depend on the dough type, e.g., Klingler (2010) used 10 g of steam per kilogram of bread, while Dessev et al. (2011) recommended 0.33–1.33 l of water per m^3^ of baking oven volume. In the case of bread rolls, Schirmer et al. (2011) used up to 3.255 l of water per m^3^ of the baking space, which resulted in the volume fraction of steam of 92%.

The simplest way to add water to the oven is by using externally produced steam or by spraying the water through a nozzle directly onto a rotating fan (Ladenbacken, 2003). Within seconds, the water evaporates and is being distributed evenly in the oven by the airflow of the fan. This approach is not suitable when a large amount of water needs to be added to the oven, or the water addition occurs in intervals.

### Novel Baking Oven Concept Based on Porous VCB

**Figure 2:**
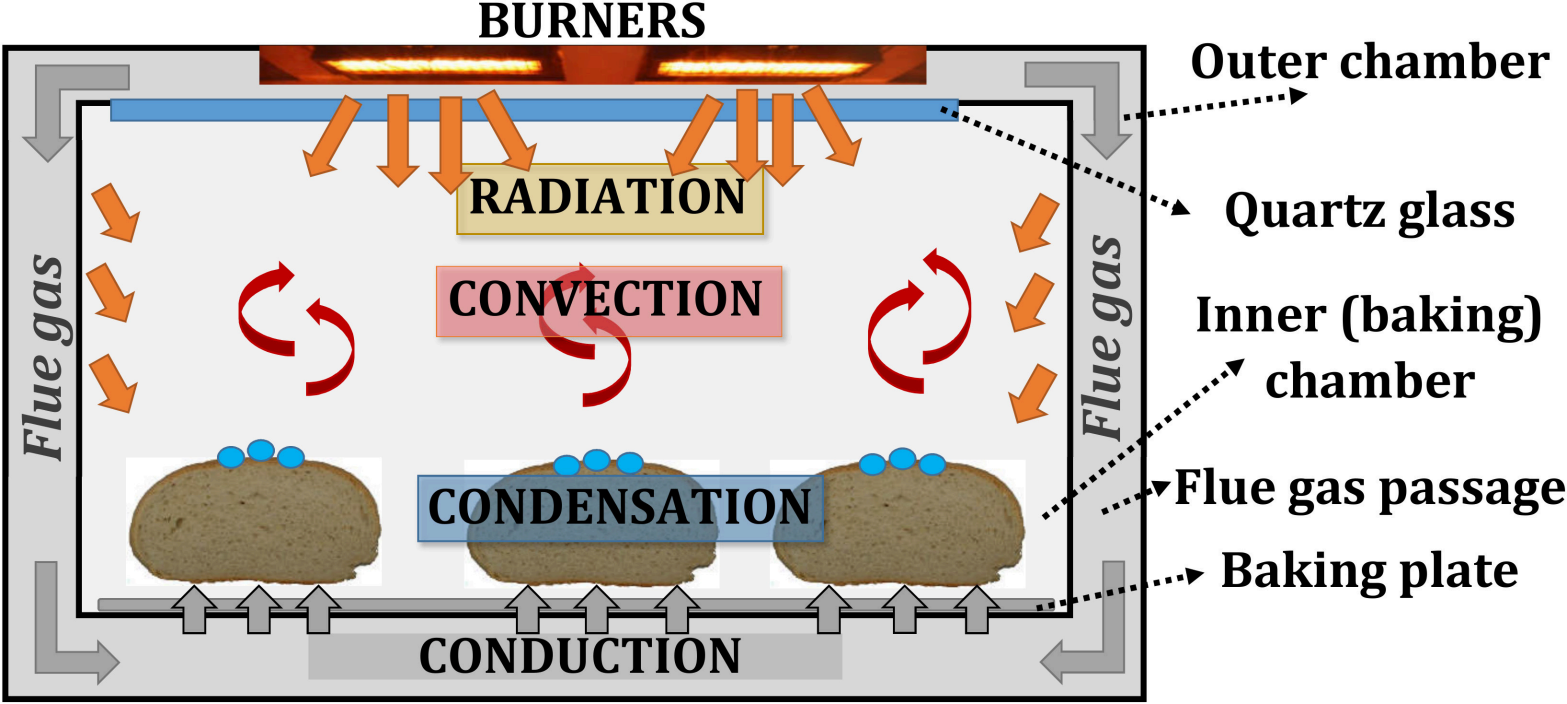
Heat transfer within a baking oven: conduction, convection, radiation, and condensation.

The gas-fired deck baking oven, based on the porous VCB technology (Jovicic and Delgado, 2018; Jovicic et al., 2019), consists of two chambers, as shown in Figure 2: (1) the inner (baking) chamber, where baking goods can be placed, and (2) the outer chamber, where porous VCBs, as heat sources, are situated and which directs the flue gas flow around the inner oven to the oven's outlet. The thermal radiation, emitted by porous VCBs, heats the inner chamber and baked goods directly, through the inner chamber's ceiling, which is made of quartz glass. Flue gas does not come in contact with the products, as the flue gas flows through the outer chamber and heats the walls of the inner chamber by convection. Consequently, both thermal radiation (from porous VCBs and the oven walls) and the convection (forced, through the flue gas flow in the outer chamber, and natural, through the movement of the baking atmosphere in the inner chamber) are used for baking. Construction of the described VCB-based oven and the unique flexibility of the heat source, i.e., VCBs, enables a limited influence on individual heat transfer mechanisms, e.g., on NIR radiation, through the temperature of the VCB surface, on convection, through the temperature and the velocity of the flue gas, and on heat conduction, through the temperature of the baking plate.

The operation of the novel baking oven was characterized in an oven prototype using 800-g white bread loaves (12 per batch) (Jovicic and Delgado, 2018). The obtained results demonstrated that this innovative concept reduces the overall baking time, both in the preheating phase and in the baking phase. As a result, a reduced fuel demand of the baking oven can be expected. Uniformity of the crust color per batch indicated that the thermal radiation flux over the baking plate was uniform. The analysis confirmed that the products possess similar sensory properties as obtained in the reference electrical deck oven, i.e., similar crust color and crumb properties, thinner crust. Further, the combination of the wide operating range and a fast response time, resulting from the VCB's operational characteristics, enables the implementation of a wide range of baking programs and allows fast adjustment to products.

### Goal

In order to successfully integrate porous VCB technology into the novel baking oven concept, it is necessary to characterize the heat transfer between the heat source and the targeted objects. The quality of baking depends, among other parameters, on the temperature level inside an oven, the homogeneity of a temperature field over the surface of baked goods, and the contribution of individual heat transfer mechanisms during baking (Schünemann and Treu, 2002; Klingler, 2010). The goal of this work was to characterize the heat transfer to a target object, placed inside a model baking oven heated by two porous VCBs, and to determine the contribution of individual heat transfer mechanisms to the total heat flow rate.

Further, the addition of water during baking is an important factor that influences product quality, but it is also connected to energy losses (Schulz, 2014). To reduce them, a better understanding of interactions, especially in terms of heat transfer, between an oven, dough, and water is needed. Using the difference method, described further in the text, the heat flow rate to the test object was separated into contributions of individual heat transfer mechanisms (heat conduction, convection, thermal radiation, and condensation). Experimental analysis was conducted without and with the addition of water to the oven atmosphere, where the form and water quantity were varied.

The knowledge, gained in the scope of this study, can be further used as a base to optimize the process parameters and to increase the efficiency of the baking process.

## Materials and Methods

**Figure 3:**
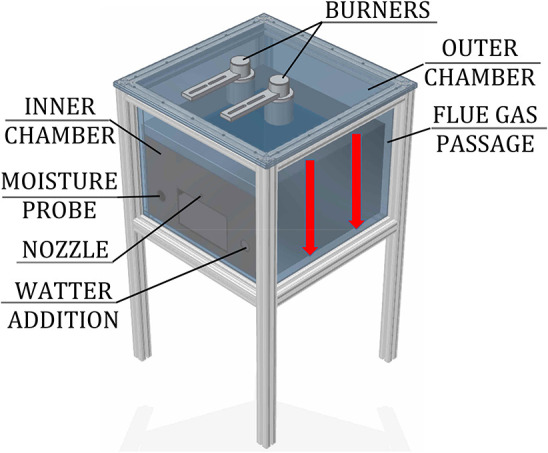
Scheme of the model oven.

The role of individual heat transfer mechanisms was experimentally determined within a model oven, shown in Figure 3, heated by two porous VCB. The concept of this model oven is the same as the novel baking oven, presented in Figure 2, but with the reduced size.

The experimental setup consisted of a model oven (inner chamber: 0.48 m × 0.48 m × 0.3 m) with two chambers (inner, for placing the test object, and outer, where the flue gas flows) and heated by two round VCBs, the water-addition unit, and the data-acquisition system. Burners consisted of a combustion zone (diameter *d* = 45 mm, height *h* = 15 mm), made of silicon-infiltrated-silicon-carbide (SiSiC), and a flame trap (*d* = 45 mm, *h* = 30 mm), produced from a perforated vacuum-formed Al_2_O_3_-ceramic plate. They were placed in the ceiling of the outer chamber, facing the test object, placed in the inner chamber. A quartz glass wall, having a high transmissivity (>94%) in the spectral range of 1–2 μm, was placed in the ceiling of the inner chamber. It provided optical access for the NIR thermal radiation from the burners to the test object, but at the same time, it prevented the flue gases from entering the inner (baking) chamber. The gap between the inner and outer chambers, in which the hot flue gases flow (red arrows in Figure 3), heating the steel walls of the inner chamber, was 10 mm wide. Outer walls of the oven were thermally insulated.

**Figure 4:**
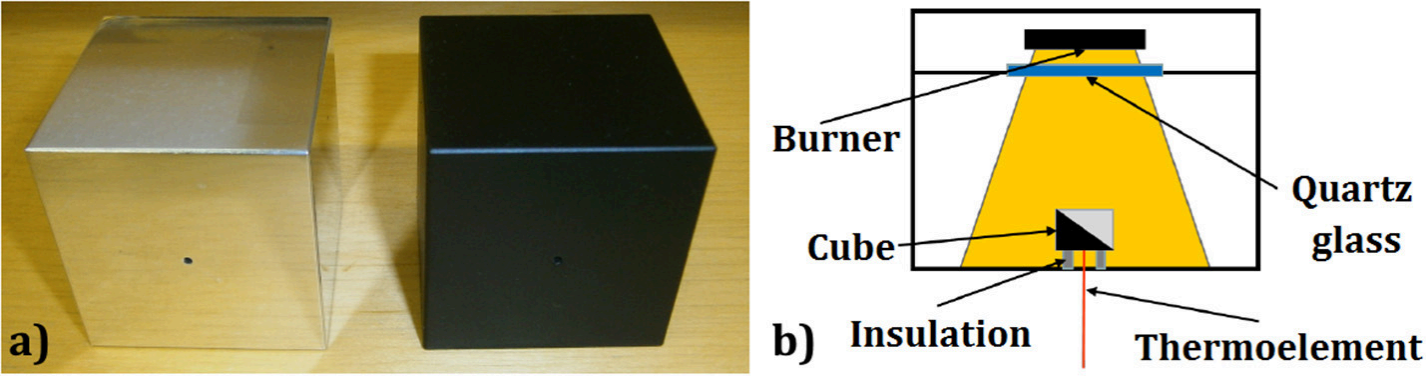
**(a)** Test objects: left—polished cube, right—black cube. **(b)** Scheme of the measurement setup.

As test objects, two aluminum cubes (*a* = 50 mm, *m* = 0,338 kg) were applied: one polished and one painted in black, as depicted in Figure 4a. The emissivity of the polished cube surface is ε = 0.039 (VDI-Wärmeatlas, 2013), but in the scope of this work it was assumed to be equal zero, i.e., the incident radiation was assumed to be completely reflected. The emissivity of the black-painted cube surface is ε = 0.97 (VDI-Wärmeatlas, 2013), but in the scope of this work it was assumed to be equal to one, i.e., the incident radiation was assumed to be completely absorbed. Due to different emissivity characteristics of the test objects, the thermal radiation mechanism of heat transfer can be filtered. Depending on the test configuration, the test object was placed directly on the bottom plate, made of aluminum, or on insulation supports, made of calcium-silicate, as shown in Figure 4b. In this way, it was possible to filter out the heat conduction through the baking plate (heat conduction from the air was neglected).

Besides thermal radiation, the heat transfer to the test object occurred by heat conduction (from the bottom plate, which was insulated from the environment) and by natural convection (through the air movement within the inner oven chamber, due to the temperature, and consequently, the density differences within it). As the two test objects have the same inner characteristics, the convection and conduction heat transfer rates were assumed equal, while the heat transfer through radiation differed due to different surface characteristics.

Heat transfer to the surface of the test object through condensation occurred in the set of additional experiments, in which the water was externally added to the oven atmosphere. The addition of water to the oven atmosphere was realized by two methods: (1) direct water spraying through a nozzle and (2) injection of steam, produced by a steam generator. In the first method, as the test object reached 30°C, the spraying of water (at 20°C), using a single-substance, full-cone nozzle, started. Water entered the inner chamber in the form of an aerosol. In the second method, steam was produced through direct contact between water and a bed of ceramic spheres, heated to 500°C. When the test object reached 30°C, water was dosed to the steam generator, evaporated, and led to the inner chamber.

In the scope of this analysis, the influence of the water addition of 25, 50, and 75 ml on the heat transfer characteristics within the model oven was investigated. Considering the baking chamber volume (~0.07 m^3^), this resulted in specific water-to-air ratios of 0.36, 0.72, and 1.09 l/m^3^, respectively.

It is important to notice that the described test method has certain limitations. One of those limitations is related to the experiments with the external addition of the water/steam into the baking chamber at the beginning of the tests. In the case of those experiments, it is not possible to clearly differentiate which part of the heat is transferred to the test object via convection and which part corresponds to the condensation/evaporation of the water from the test object. From the moment of the water/steam injection until all previously condensed water evaporates from the surface of the cube (condensation and evaporation zones described later on in the chapter 3.2.), the applied methodology allows only quantification of the joint effect of these two, essentially different heat transfer mechanisms (convection and condensation/evaporation).

### Measurement of Heat Transfer in Literature

Heat flux within an oven can be measured using temperature probes with known physical properties, i.e., heat flux sensors. Krist-Spit and Sluimer (1987) characterized heat transfer in an indirectly heated oven, where the radiation share dropped from 73 to 59% as the air velocity within the oven increased. Similarly, Fahloul et al. (1994) determined that the thermal radiation share within a continuous, indirectly heated gas oven decreased from the starting value of 49% (air temperature 200°C, low airflow) as the airflow increased. Zareifard et al. (2009) measured the heat flux within an electrical oven, where different heat transfer mechanisms could be altered, e.g., thermal radiation was suppressed by wrapping the heat flux sensor in a reflective aluminum foil. Depending on the air velocity and temperature, the radiation share within the oven was in the range of 63–89%.

Shibukawa et al. (1989) used heat flux sensors in the form of copper cylinders with known emissivities to characterized heat transfer in a furnace, where the ratio of the radiative to the total heat transferred was about 70% at the oven air temperature of 200°C. Similarly, Altomare (1994) applied aluminum blocks with only one side exposed to heat transfer.

### Measurement and Control

The heat flow rate between the baking oven and the test object was determined by measuring the temperature at different positions in the oven, using K-type thermocouples: bottom plate temperature in the plate's centrum below the test object, the temperature in the centrum of the test object, flue gas temperature in the flue gas duct, and air temperature in the inner chamber at the level of the test object. Relative humidity was measured using a high-temperature hygrometer Hygrophil Z Type 1701-41 (Co. Bartec). Its probe tip was placed at the center of the inner chamber. The measurement error of this device is 1 _Vol_% H_2_O. The flow rates of the fuel gas (methane) and the combustion air to the burner were independently controlled, using mass flow controllers. Measured data (relative humidity, chamber atmosphere temperature and cube temperature) were collected by a Data Acquisition System, based on the LabVIEW Software.

### Measurement Procedure

Tests started with igniting the burners (total power *P* = 1.5 kW, λ = 1.3) to heat up the oven. When the initial steady state was reached, the burner power was adjusted to *P* = 1.3 kW (0.65 kW per VCB, i.e., 400 kW/m^2^ of the VCB active surface), to reach the target temperature of the bottom plate of ~230°C (without water input). This temperature was chosen as a standard temperature of the bottom plate for the production of many baked goods (MIWE, 2011), although for ovens with high levels of thermal radiation a lower oven temperature (~180°C) is also possible (Ploteau et al., 2015). When the steady state in terms of the bottom plate temperature was reached, the test cube (conditioned to 4°C) was placed in the center of the oven, below the burners. Measurements started when the temperature in the cube centrum reached 30°C and lasted 30 min in order to ensure that the test cubes reached the temperatures higher than the crust of the baking goods would under the same baking conditions. At the end of the process, the test object was removed from the oven. When the influence of water present in the oven atmosphere was analyzed, water addition started simultaneously with the start of the measurements. Evaluation of the heat exchange between the burners and the test object was conducted by analyzing the temperature curves of the individual arrangements at a certain relative humidity level, according to the procedure described below. An overview of the conducted measurements is given in Table 1.

**Table 1:** Summary of the conducted measurements.

	**Water (ml)**	**Arr**.	**Average**	
			**Plate temp. [^**°**^C]**	**Vol% H_**2**_**
Zero water	0	*BCP*	221	3.02
		*BCI*	225	3.57
		*PCP*	223	3.42
		*PCI*	223	3.64
Water spraying	25	*BCP*	220	33.7
		*BCI*	217	35.5
		*PCP*	220	35.3
		*PCI*	218	34.4
	50	*BCP*	212	50.0
		*BCI*	213	49.6
		*PCP*	217	52.9
		*PCI*	217	49.9
	75	*BCP*	206	61.0
		*BCI*	207	60.4
		*PCP*	214	60.8
		*PCI*	211	60.7
Steaming unit	25	*BCP*	225	39.1
		*BCI*	222	37.0
		*PCP*	222	40.1
		*PCI*	221	36.3
	50	*BCP*	222	55.1
		*BCI*	221	55.7
		*PCP*	221	54.5
		*PCI*	222	60.6
	75	*BCP*	223	62.7
		*BCI*	222	66.7
		*PCP*	222	63.9
		*PCI*	220	65.8

### Heat Transfer Calculation

In this work, the difference method was used to determine the contribution of individual heat transfer mechanisms, i.e., heat conduction (HC), convection (C), thermal radiation (TR), and condensation (Cond). The difference method, as used in this work, is based on the approach developed at the University of Applied Sciences Bremerhaven, under Prof. Lösche (Büsing, 2011; Börsmann et al., 2015).

Its principle is based on the measurements of the heat flow rate to the two test objects (polished and black-painted copper cubes), placed inside an oven. In order to alter different heat transfer mechanisms, two test objects were arranged in four configurations, i.e., placed either directly on the oven's bottom plate or on the insulation supports. The four test object arrangements, also applied in this work, are overviewed in Table 2. Heat flow rates were analyzed as a function of time (*Q* = *f* (*t*)) and as a function of the oven's temperature settings.

**Table 2:** Experimental test configurations of the difference method.

**Arrangement**	**Scheme**	**Heat flow rate**
**PCI**—**P**olished **C**ube on the **I**nsulation layer	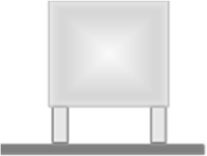	*Q_*PCI*_ = Q_*C,PCI*_ + Q_*Cond,PCI*_*
**PCP—P**olished **C**ube on the baking **P**late	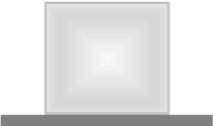	*Q_*PCP*_ = Q_*HC,PCP*_ + Q_*C,PCP*_ + Q_*Cond,PCP*_*
**BCI—B**lack painted **C**ube on the **I**nsulation layer	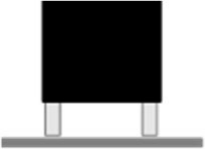	*Q_*BCI*_ = Q_*C,BCI*_ + Q_*TR,BCI*_ + Q_*Cond,BCI*_*
**BCP—B**lack painted **C**ube on the baking **P**late	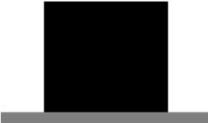	*Q_*BCP*_ = Q_*HC,BCP*_ + Q_*C,BCP*_ + Q_*TR,BCP*_ + Q_*Cond,BCP*_*

First, the temperature range of interest is divided into narrow intervals, as the temperature difference between the cube and the surrounding is the driving force for the heat transfer. The average heat flow rate, *Q* within each interval can be determined as

(1) $$\begin{array}{l} Q=\frac{m_{Cu}\cdot c_{p,Cu}\cdot \Delta T}{t} \end{array}$$

where *m*_*Cu*_ is the mass of the cube, *c*_*p,Cu*_ is the specific heat capacity of the cube, Δ*T* is the temperature difference between the cube's center at the beginning and the end of the process, and Δ*t* is the time interval.

The share of individual heat transfer mechanisms can be calculated as the difference between different test object configurations. The contribution of individual heat transfer mechanisms to the heat flow rate in these configurations was calculated assuming following simplifications:

• *PCI* (Polished Cube on the Insulation layer) exchanges heat only through natural convection with the surrounding inner–chamber atmosphere. Heat conduction from the bottom plate and from the surrounding oven atmosphere was assumed negligible (cube placed on insulated supports, and heat conductivity of air is low). Heat exchange via thermal radiation was neglected since the cube's surface was assumed perfectly reflective. Thus, the heat flow rate by convection was assumed to be equal to the total heat flow rate, i.e., *Q*_*C,PCI*_ = *Q*_*PCI*_.

• *PCP* (Polished Cube on the baking Plate) exchanges heat through heat conduction with the bottom plate and through natural convection with the inner chamber atmosphere. Assuming that the heat exchange via convection remains constant for each test configuration, *Q*_*PCI*_ and *Q*_*PCP*_ were combined to determine the heat conduction component of the heat flow rate:

(2) $$\begin{array}{l} Q_{HC,PCP}={Q_{PCP}-Q_{C,PCP}=Q}_{PCP}-F\cdot Q_{PCI}\\ \begin{array}{l} \;=Q_{PCP}-\frac{5}{6}\cdot Q_{PCI} \end{array} \end{array}$$

where *F* is the convection correction factor, which corresponds to the number of cube sides that participate in heat transfer (*PCI*-−6 sides, *PCP*−5 sides, since the cube was placed directly on the bottom plate).

• *BCI* (Black painted Cube on the Insulation layer) is assumed to exchange heat only through natural convection and thermal radiation. Under this assumption, the heat transferred to the BCI cube by thermal radiation could be directly determined, since the convection component of *BCI* is equivalent to *PCI*:

(3) $$\begin{array}{l} Q_{TR,BCI}=Q_{BCI}-Q_{PCI} \end{array}$$

•*BCP* (Black painted Cube on the baking Plate) is assumed to exchange heat by all three heat transfer mechanisms, which are determined as

(4) $$\begin{array}{l} Q_{HC,BCP}={Q_{BCP}-{F\cdot Q}_{BCI}=Q}_{BCP}-{\frac{5}{6}\cdot Q}_{BCI} \end{array}$$

(5) $$\begin{array}{l} Q_{TR,BCP}=Q_{BCP}-Q_{PCP} \end{array}$$

(6) $$\begin{array}{l} Q_{C,BCP}=Q_{BCP}-\left(Q_{HC,BCP}+Q_{TR,BCP}\right) \end{array}$$

In the scope of the here presented work, the above-described difference method was slightly modified (Burjakow, 2016):

- Aluminum cube (Figure 4a) was used instead of copper, as it was expected that the aluminum surface would better preserve its low emissivity in the oxidizing atmosphere. Cube has a uniform 3D temperature field equal to the core temperature, since the Biot number for this cube is Bi = 0.0095 (<<1) for natural convection and Bi = 1 for film condensation.
- Calculation of heat transfer through heat conduction in the configuration *BCP* was modified to
  (7) $$\begin{array}{l} Q_{HC,BCP}=Q_{BCP}-{\frac{5}{6}\cdot Q}_{C,BCI}-\frac{11}{12}\cdot Q_{TR,BCI} \end{array}$$
  The factor 5/6 was assigned to the convective heat flow rate, as was explained previously. For the heat transfer through thermal radiation, the factor 11/12 was assumed; in the *BCI* configuration, the heat can be transferred by thermal radiation also to the cube's bottom (from the bottom plate), but as it is not exposed directly to the burner, the factor was reduced from unity.
- Beside the analysis of the heat flow rate as a function of time, an attempt was made to analyze the heat transfer based on the cube temperature, i.e., *Q* = *f* (*T*_*Cu*_). In different cube configurations, different temperatures, and thus different driving forces, were reached at the same time points of measurements, thus it was estimated that the cube temperature would be a more suitable reference for the detailed results' analysis.
  To obtain the function *Q* = *f* (*T*_*Cu*_), measured cube temperatures, as discrete values, needed to be transformed to a continuous function of time. In this way, a heat flow rate in a certain temperature range could be evaluated and connected to the flow rates in other arrangements. A ninth-degree polynomial function of the cube temperature, *T*_*Cu*_ with time, t was expressed as
  (8) $$\begin{array}{l} t\left(T_{Cu}\right)=a_{0}+\overset{9}{\underset{n=0}{\sum }}a_{n}\cdot T_{Cu}^{n} \end{array}$$
  where *a*_*o*_ – *a*_*n*_ are the weights of the polynomial regression.

## Results and Discussion

### Oven Temperature

During the experiment, the average air temperature in the inner chamber (measured at the level of the test object without water addition) was ~223.9 ± 3.6°C (reached ca. 2 min after the test object was inserted). When water aerosol was injected into the baking chamber through a nozzle, the average oven temperature decreased up to 14°C, depending on the amount of injected water. The stable air temperature was again reached ca. 7 min after the test object was inserted. The injection of steam had no significant influence on the average baking chamber air temperature.

### Cube Temperature Changes

**Figure 5:**
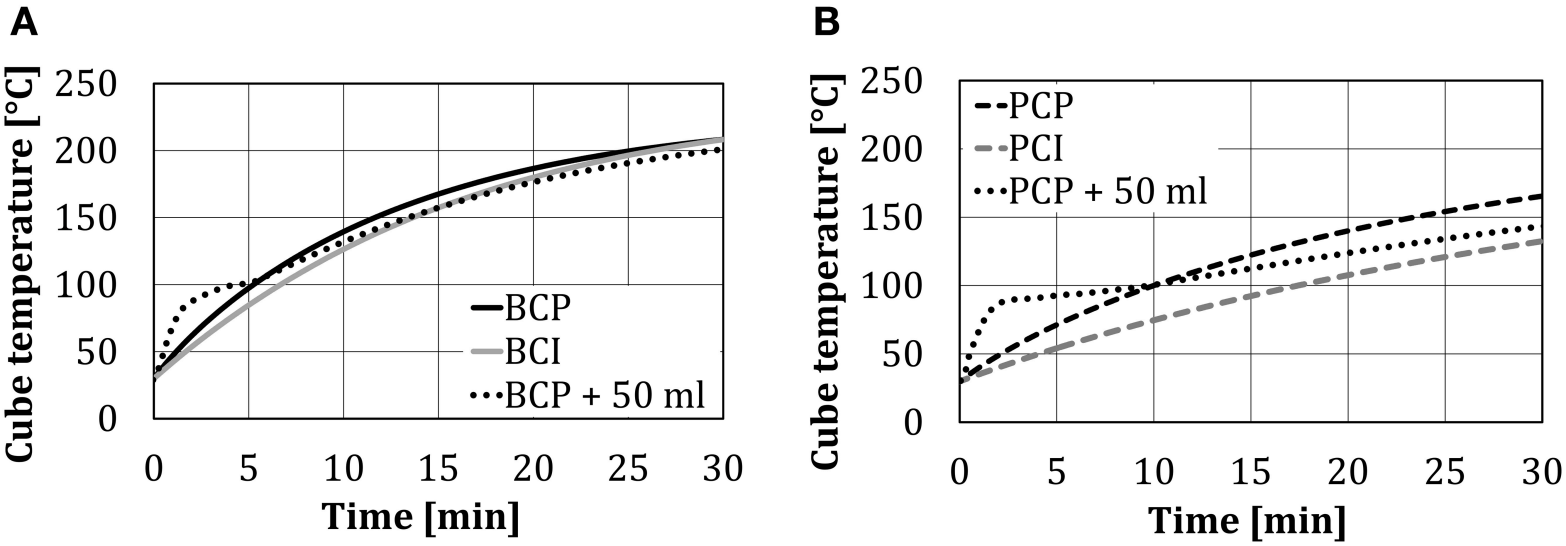
Cube temperatures without/with 50 ml of water spray injection at different test object arrangements: **(A)** black cube, and **(B)** polished cube.

The change of the cube temperatures for four test arrangements (without and with 50 ml of water spray injection) is presented in Figure 5. As was expected, the temperature in the center of the black cube (*BCI, BCP*) was higher compared to the temperature of the polished one (*PCI, PCP*), due to the additional heat transfer by thermal radiation. At the end of the 30-min test period, the temperature in the center of the black cube was ~30°C higher compared to the polished one. A very small temperature difference between *BCP* and *BCI*, as shown in Figure 5A, indicates that the heat transfer through heat conduction (from the bottom plate) was negligible compared to heat transfer through thermal radiation.

Changes in the cube core temperature in time, in the case of the cubes placed directly on the plate (*PCP* and *BCP*) and on the insulating support (*PCI* and *BCI*), differed significantly in the first 10 min (evaluated results—temperatures relevant for the real baking process) and afterward were approximately the same. This occurred as the heat transfer through heat conduction significantly declined after some time (~10 min), as the cube temperature approached the plate temperature.

**Figure 6:**
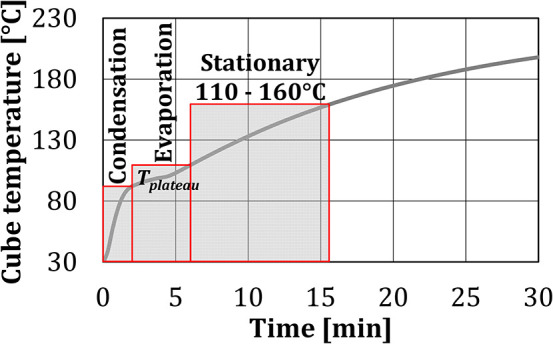
Temperature zones for *BCP* at water addition of 75 ml by the nozzle.

When water spray was injected into the baking chamber, the shape of the temperature curves in the first 5 min of the experiment changed. These results indicated that the test period of 30 min can be divided into three characteristic zones, where different heat transfer mechanisms were dominant. Since the heat transfer depends on the temperature difference, the limits of each zone are associated with the temperature of the test object, as shown in Figure 6.

*Condensation zone* (*T*_*C*_ = 30°C – *T*_*plateau*_) refers to the zone of the highest temperature gradient, where part of the injected water spray/steam was initially condensed on the cold surface of the test cubes, as the cube surface temperatures were below the dew-point temperature. The cube temperature increased sharply, both due to heating by the burners and due to the transfer of latent heat of water to the cube. In this zone, joint effects of the convective heat transfer and heat transfer due to the condensation of the water on the surface of the test object are measured together. *Evaporation zone* (*T*_*C*_ = *T*_*plateau*_ – 110°C) covers a nearly constant temperature plateau, where the water condensation on the cube surface and evaporation from it are in equilibrium. In this zone, joint effects of the convective heat transfer and heat transfer due to the evaporation of the water from the surface of the test object are measured together. The transition temperature between the condensation and the evaporation zones, identified as the change in the temperature gradient from steep to very mild, differed between the experiments as the equilibrium depends on the air temperature and the relative humidity. As the end temperature of the evaporation zone, 110°C was chosen, to insure that the water evaporation from the cube surfaces is completed. *Stationary zone* (*T*_*C*_ = 110–160°C) is the zone of slow temperature increase between 110 and 160°C, where evaporation and condensation no longer take place. The end temperature of 160°C was chosen, as according to Ahrné et al. (2007) this is the maximum crust temperature of bread at the air temperature in the baking chamber of 220°C.

### Influence of Quantity of Added Water

**Figure 7:**
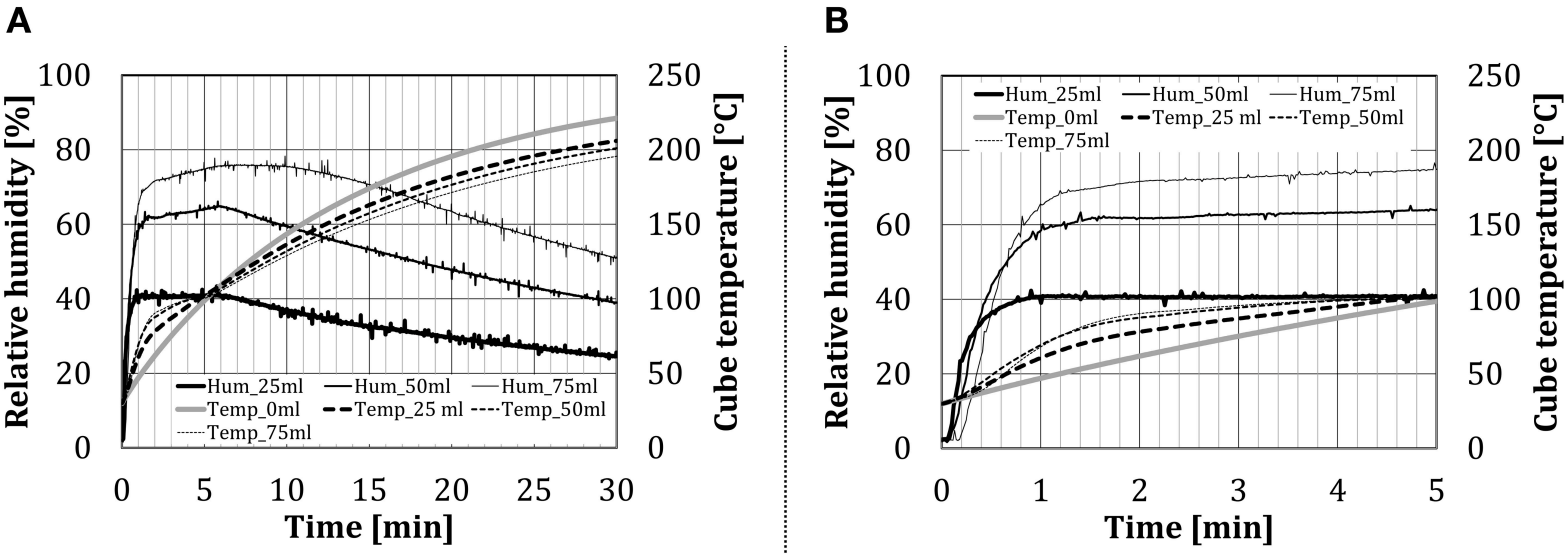
Time dependency of the relative humidity for the configuration *BCP* at different water amount (addition by the nozzle): **(A)** over the whole test period and **(B)** first 5 min in details.

Figure 7 shows the time change of relative humidity inside the inner chamber and of the temperature in the centrum of the test object in the configuration *BCP*, for different amounts of water spray injected through the nozzle. Results show that the relative humidity in the oven increased almost instantaneously (within 1 min, depending on the amount of water added), to reach a certain steady value. The test object temperature increased steeply for ~2 min, which corresponds to the previously mentioned condensation zone. After that, the temperature gradient started to decline, due to increased water evaporation from the cube surface, which can be noticed as a mild increase in the relative humidity curve. Schirmer et al. (2011), which used baked goods as test objects, described a similar behavior at the beginning of their experiments. After ~5 min from the beginning of the experiment, a very slow decline in the relative humidity follows. In the baking experiments (Schirmer et al., 2011), the decrease did not occur, as the constant evaporation from the moist dough stabilizes the relative humidity value.

### Heat Flow Rate to the Test Cubes (No Water Addition)

In this section, the heat flow rate to the test cubes without the addition of water will be discussed. When water spray/steam was not injected into the baking chamber, the heat transfer can be analyzed from the beginning of the experiments.

**Figure 8:**
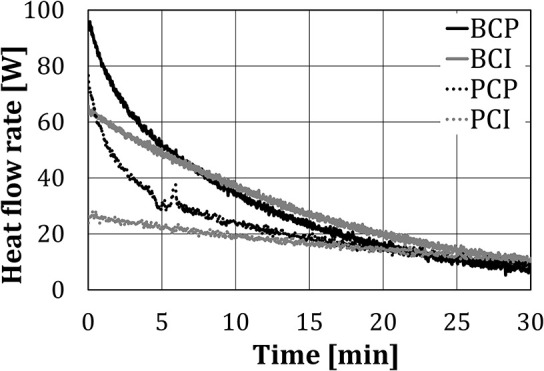
Heat flow rate to the cubes, without water spray/steam injection.

Figure 8 shows the heat flow rate, calculated using Equation (1), for different test configurations. The heat flow rate for *BCI* and *PCI* decreased approximately linearly with time, while for *BCP* and *PCP* a polynomial reduction rate can be noticed.

**Figure 9:**
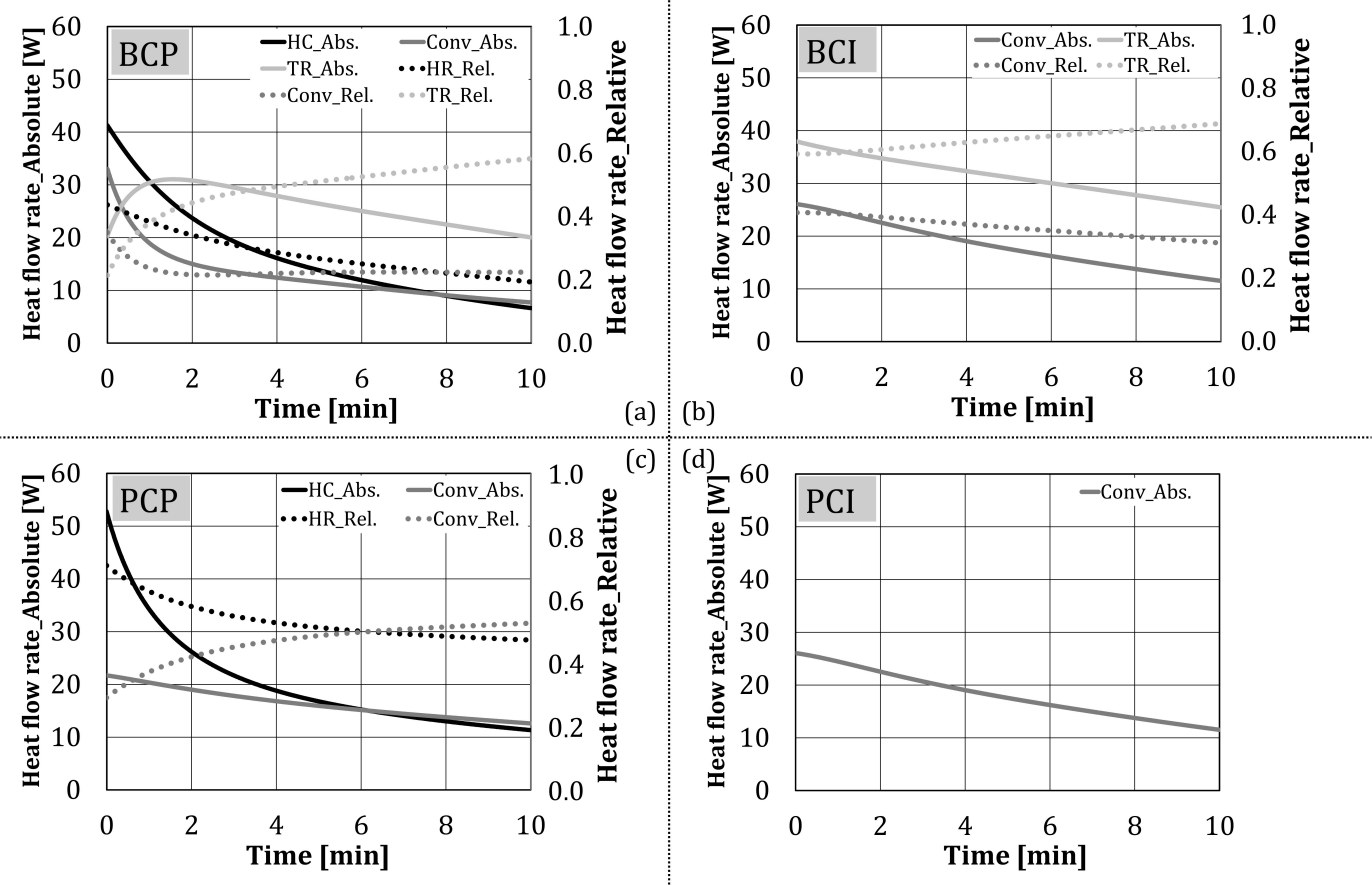
Absolute and relative shares of heat transfer mechanisms for four test configurations (no water addition).

Figure 9 summarizes the absolute and relative shares of heat transfer mechanisms (heat conduction, convection, and thermal radiation) for four test configurations, determined using Equations (3)–(5). Results were presented in the form of time dependency, for the first 10 min of the experiments. In this time, the temperature of the cubes corresponds to the temperatures of the baking goods in the real process. Figure 9 shows that the heat flow rate per each mechanism mainly decreased with time for each tested configuration. The shape of thermal radiation dependency on time differed between *BCI* and *BCP* in the first 2 min of the experiment, as thermal radiation for these two configurations were calculated separately, using Equations (4) and (5), respectively. After the second minute, the thermal radiation components of two configurations had approximately the same value. Regarding the relative shares, the heat conduction share decreased rapidly at the beginning of tests, as the temperature of the test object and the plate approach. The share of thermal radiation increased with time, as its absolute value decreased slower compared to the other two heat transfer mechanisms. Also, as the experiments proceed, besides the main source of radiation, i.e., two VCBs having the surface temperature of ~900°C (Keramiotis et al., 2012), the thermal radiation was additionally emitted by the oven walls (T = 200–400°C with the emissivity of 0.2–0.3 (MIWE, 2011).

In case of *PCP*, the heat flow rate through heat conduction (through the baking plate) decreased faster compared to convection, indicating the behavior of driving forces, i.e., temperatures of the bottom plate and the test cube approached faster compared to the temperatures of the oven atmosphere and the test cube.

**Figure 10:**
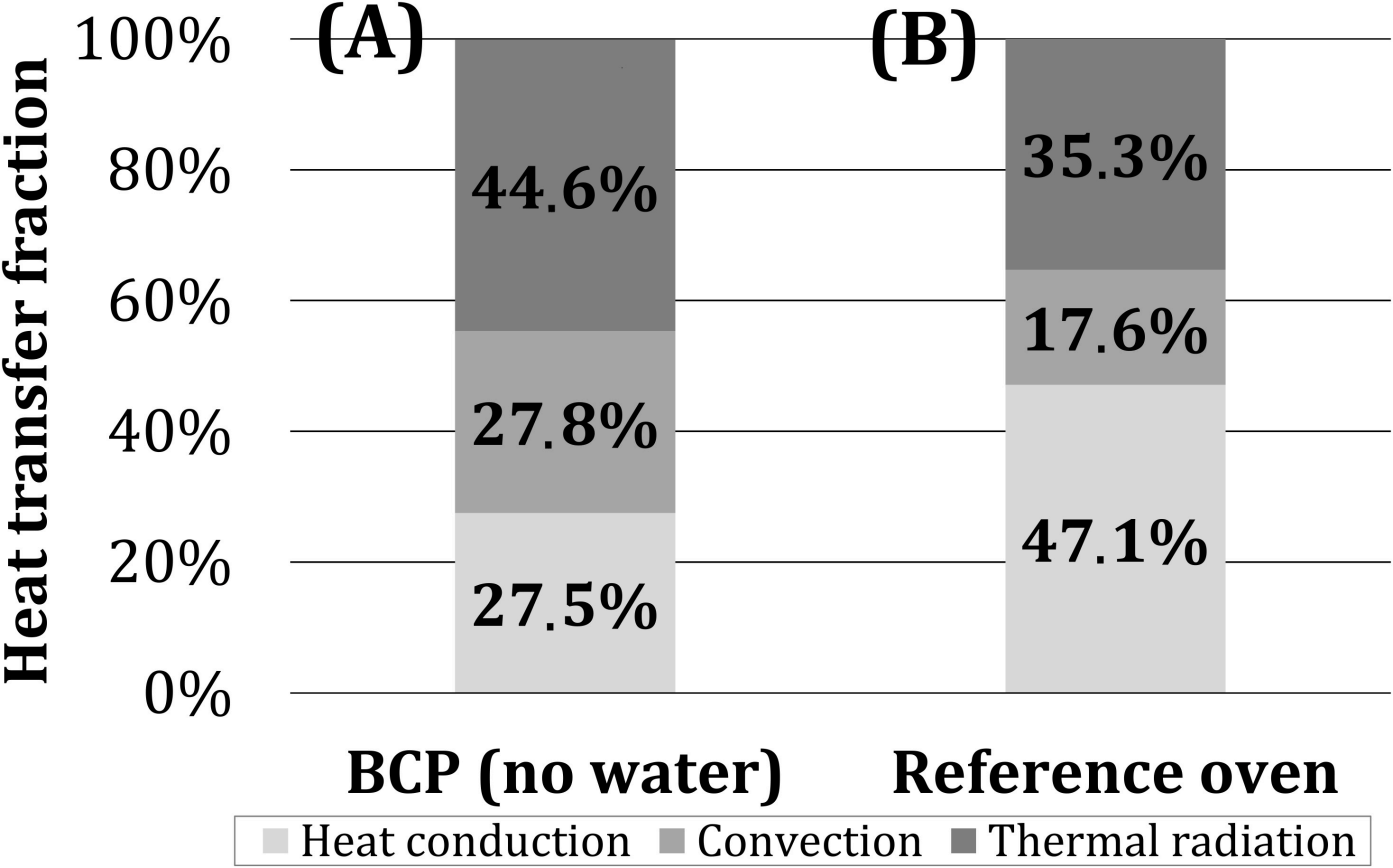
Role of individual heat transfer mechanisms for **(A)** *BCP* (after 10 min, without water addition) and **(B)** an industrial deck oven (MIWE, 2011).

In Figure 10, the shares of heat transfer mechanisms for *BCP* in the first 10 min of the experiment were summarized and compared to the commercial deck oven (MIWE, 2011). Results, obtained in the scope of this study, indicate that the dominant heat transfer mechanism (in the cube temperature range corresponding to the real baking good temperatures—first 10 min of the tests) in the model oven, heated by two porous VCBs, was thermal radiation, with the share of ~45%. The shares of heat conduction and convection were determined to be ~27.5%. In the reference deck oven, which is heated electrically, heat conduction is the dominant heat transfer mechanism. One of the possible reasons for this, besides the difference in the heat source characteristics, can be that the baking plates in the reference oven are made of glass-fiber-reinforced cement, having a higher heat storage capacity than the steel bottom plate of the model oven (Safety Data Sheet, 2010; VDI-Wärmeatlas, 2013). The comparison presented in Figure 10 should be taken only as a rough indicator, as the contribution of individual mechanisms to heat transfer is affected not only by the heating method but also by the oven structure, material, and geometry (Baik et al., 1999; Ploteau et al., 2015).

### Heat Flow Rate to the Test Cubes (With the Injection of Water)

When the water spray was injected into the baking chamber, it evaporated in the hot oven atmosphere and partially condensed on the test object, thus transferring the latent heat to the object. As the temperature of the cube increased, due to heating by the burners, the initially condensed water started to evaporate, thus transferring the heat back to the oven's atmosphere. As was expected, when the water was added in the form of aerosol by the nozzle, the total heat flow rate to the test object was lower compared to that of the steam addition, due to the evaporation.

As is shown in Figure 6, the heat transfer to the cube, when water was externally added to the oven atmosphere, could be related to the cube temperature. When different test configurations are to be related, the cube temperature seems like a more accurate parameter, as different temperatures in the cube were reached at the same time points of the experiments. This led to different temperatures to the environment and thus to different driving forces for heat transfer. Depending on the cube temperature, heat transfer mechanisms were analyzed separately in three zones, analog to Figure 6: condensing, evaporation, and stationary zone.

#### Condensing Zone

**Figure 11:**
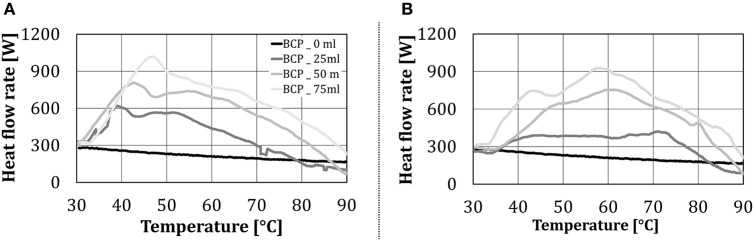
Heat flow rate to *BCP* in the condensing zone, as a function of the test object temperature and the amount of added water (in case of water addition): **(A)** by the nozzle and **(B)** by the steaming unit.

During baking, additionally to heat transfer through heat conduction, convection, and thermal radiation, heat is being transferred to the surface of the product (in this work, on the surface of the test object) through condensation of water vapor. The heat flow rate in the condensing zone, as a function of the cube temperature and the addition of water (water quantity, type of addition) for the *BCP* configuration, is shown in Figure 11.

The condensing zone starts at the cube temperature of 30°C when according to the measurement procedure the injection of water spray/steam started. When water was not added to the baking chamber, the heat flow rate decreased linearly with the temperature of the cube. When the water was added to the oven, a steep increase in the heat flow rate was noticed, up to the cube temperature of ~40°C. This steep increase in heat transfer can be attributed to the heat transfer by condensation. As the cube temperature raised, the increment of the heat flow rate to the test object slowed down, and from ~50°C started to decline. The condensing zone ended at the cube temperature at which the heat flow rate with water addition fell below the one without water addition. This occurred at the intersection of the heat flow rate lines without and with water addition, which can be identified in Figure 11 to be between ~80 and 90°C (depending on the amount of added water). After this point, the heat transfer from the test object due to water evaporation exceeded the heat transfer to the test object due to condensation, i.e., condensation stopped, indicating that the surface temperature of the object exceeded the dew point (relevant for this work ~94°C at 83 _Vol_% H_2_O). Additionally, the form of the heat flow rate line for two types of water addition (aerosol by the nozzle, steam by a steaming unit) to the oven did not differ significantly.

#### Evaporation Zone

Regarding Figure 6, the evaporation zone is the zone of a low temperature gradient. As the temperature of the cube increased due to heating by the burners, the initially condensed water started to evaporate, thus transferring the heat away from the test object, i.e., the heat for evaporation (equal to evaporation enthalpy) was provided by the cube. The nearly constant cube temperature was a result of the equilibrium between the heating by the burners and the water evaporation. In the scope of this work, the end of the evaporation zone was set to be at 110°C, where complete evaporation was assumed. Exact performance curves in this zone could not be established, due to the selected method of the polynomial fitting of the measured values within this temperature range, i.e., the temperature profile is discontinuous, and such analysis leads to unreliable results.

#### Stationary Zone

**Figure 12:**
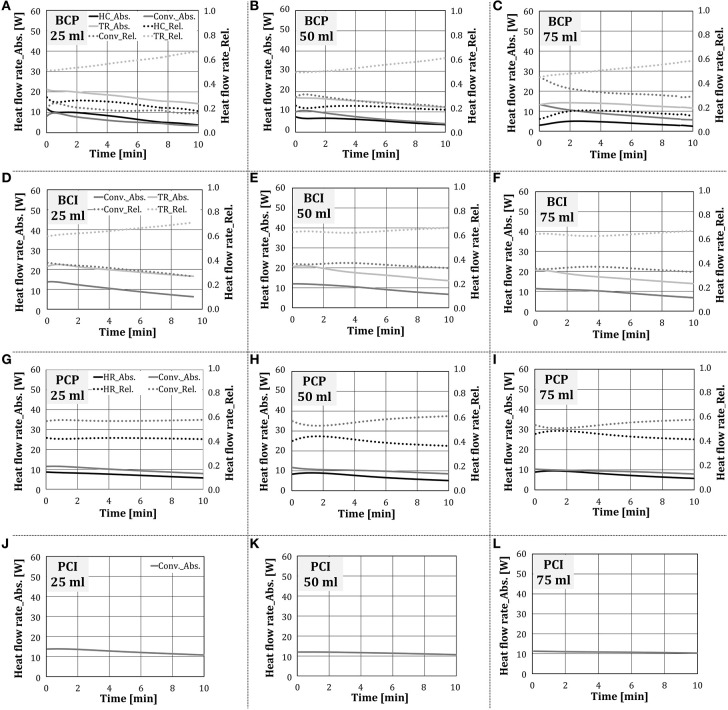
Absolute and relative shares of heat transfer mechanisms for the configurations **(A–C)** BCP, **(D–F)** BCI, **(G–I)** PCP and **(J–L)** PCI in the stationary zone (water addition through a nozzle).

In this section, the influence of the amount of added water on the heat transfer within the oven without the occurrence of condensation and evaporation was investigated. In the scope of this work, the end temperature of the stationary zone was set to 160°C. Figure 12 presents the absolute and relative heat flow rate for individual heat transfer mechanisms over time at different amounts of externally added water. The difference in the duration of individual tests corresponded to the difference in heat transfer characteristics, due to which the test object reached 160°C at different times. As no qualitative difference between two water addition methods was noticed, only the results for water addition by the nozzle were showed.

As the experiments proceeded, i.e., the cube temperature increased, the absolute values of individual heat transfer mechanisms decreased, independent on the test configuration. This finding corresponds to the situation with no water addition, shown in Figure 9. In test configurations where thermal radiation was relevant for heat transfer (i.e., *BCP, BCI*), it was the dominant heat transfer mechanism, whose relative share increased as experiments proceeded. This behavior is analogous to the situation with no water addition.

As the amount of added water raised, the heat exchange through thermal radiation to the test object decreased, although its relative share stayed approximately the same. Hottel (1927) showed that the absorption of thermal radiation by the chamber gas is, among other parameters, dependent on the water vapor partial pressure. It can be concluded that, as could be expected, water vapor absorbed part of the thermal radiation, emitted by the porous VCBs. As the amount of added water increased, the heat transfer through heat conduction decreased for *BCP* and stayed approximately the same for *PCP*. The reason for this could be the higher temperature difference between the plate and the cube in case of *PCP*, which was colder, as thermal radiation was eliminated. In this way, the driving force for heat conduction in the case of *PCP* was higher compared to *BCP*.

The convective heat transfer stayed approximately constant for the configurations *BCI, PCP*, and *PCI*, while for BCP it increased slightly. As the amount of added water increased, the temperature and the thermal capacity of the gas within the oven increased. It is to be expected that as more water was added to the oven, the test object temperature would decline, the temperature difference between the test object and the gas temperature would raise, and thus the convective heat transfer would increase.

**Figure 13:**
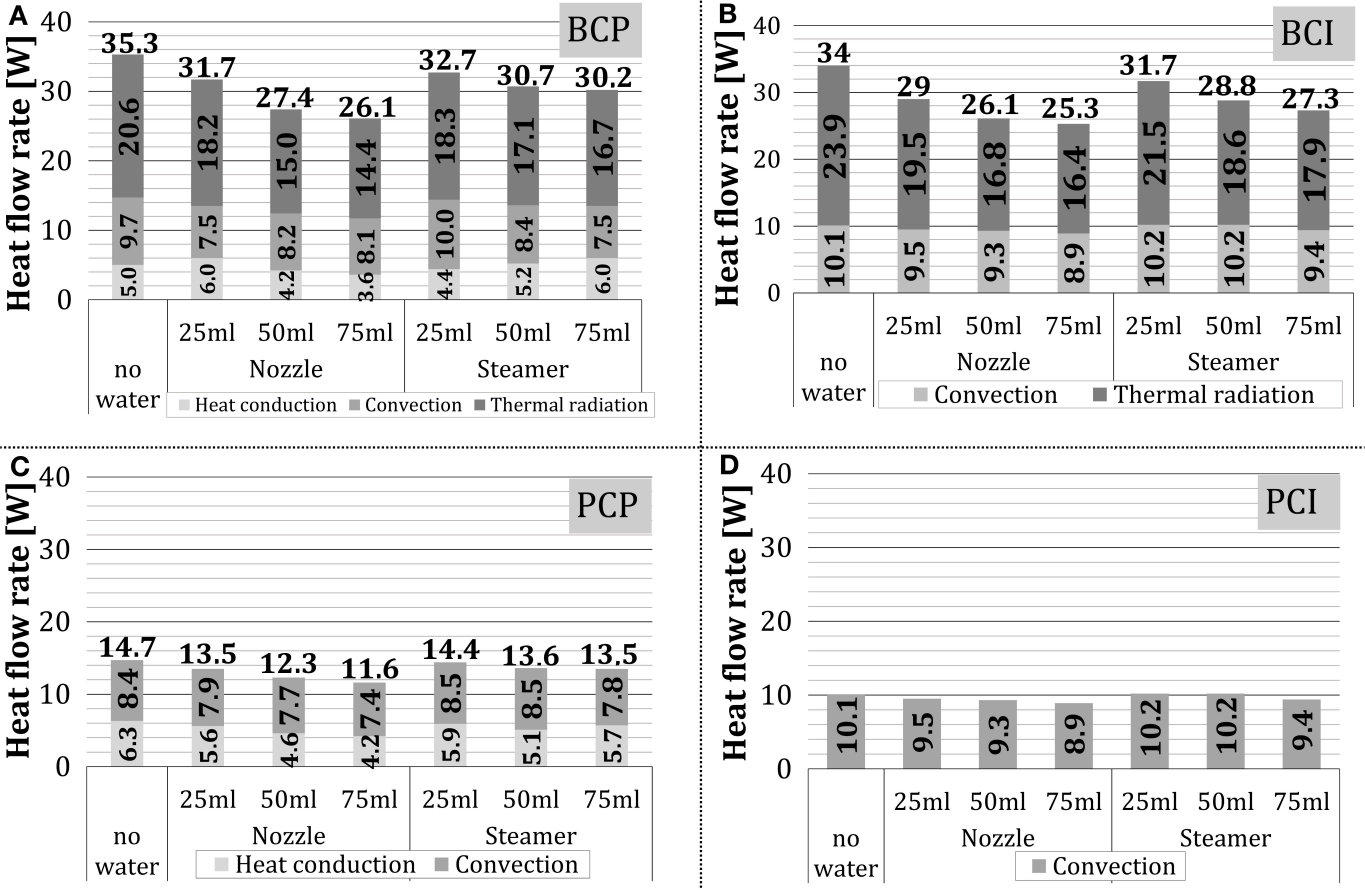
Average and relative shares of heat transfer mechanisms in the stationary zone: **(A)** *BCP*, **(B)** *BCI*, **(C)** *PCP*, and **(D)** *PCI*.

The impact of water addition to the oven on the total heat transfer and its components over the complete stationary zone is summarized in Figure 13. The presented results confirm the findings from Figure 12. Regarding the total heat flow rate, it decreased slightly with the increasing amount of added water, which was more pronounced when the nozzle was used (i.e., 25% and 31% for steam unit and the nozzle, respectively).

### Measurement and Model Error

Real emissions of the polished and the black-painted aluminum cubes deviate from these assumed, ideal values (0 and 1, respectively) and may additionally change during the experiments. The formation of an oxide layer on the aluminum surface within the moist, warm atmosphere can increase the emission coefficient from 0.039 to 0.11 (VDI-Wärmeatlas, 2013), which could directly influence the heat transfer by radiation. Further, the layer of black paint acts as an additional thermal layer between the cube and the surroundings, which can affect heat conduction.

Measurement error can be further induced by assuming that the heat conduction to configurations *BCI* and *PCI* was eliminated by application of calcium-silicate supports, which is not entirely correct. This error is however very low, as only app. 16% of the cube surface is in contact with the supports and the thermal conductivity of calcium-silicate is very low (VDI-Wärmeatlas, 2013).

Finally, it was assumed that the convective component of heat transfer is the same for the tested configurations. This assumption can be a source of error, as the temperature of the cube surface differs during experiments for different test configurations; thus, the driving force for heat transfer by convection is also different.

## Conclusions

The goal of the presented work was to characterize the heat transfer to the test objects, placed within a model baking oven, heated by two porous volumetric ceramic burners, with and without external addition of water to the chamber. The role of individual heat transfer mechanisms was determined by the difference method, using four test object configurations to alter different heat transfer mechanisms.

When water was not added to the chamber, the temperature in the centrum of the test object steadily increased with time for each configuration. When water was added in form of an aerosol or as steam, the temperature curves showed three characteristics zones. These zones were correlated with the cube temperature: (1) condensation zone, where at the lower temperature end the heat was transferred largely by condensation of water on the surface of the relatively cold cube; the cube temperature increased steeply, (2) evaporation zone, where the condensed water evaporated, thus the heat was transferred from the test object; due to the cube heating by the burners and its cooling due to evaporation, its temperature stayed approximately constant, and (3) stationary zone, where the heat transfer was affected by water presence in the inner chamber atmosphere, without the influence of condensation or evaporation.

With no water addition, the dominant heat transfer mechanism was thermal radiation (~45%), followed by heat conduction and convection (~27.5% each). During the heating process, the heat flow rate for all test configurations declined, with the steepest fall for the configurations when the test object was placed directly on the bottom plate (*BCP, PCP*). This occurred due to a fast decline in the heat conduction share, as the cube's bottom temperature reached the temperature of the bottom plate, which was made of metal with relatively low thermal capacity.

When water was added to the oven atmosphere, the heat flow rate to the test object declined. The decline increased as the amount of added water raised because the water in the oven atmosphere absorbed part of thermal radiation. Regarding the heat transfer mechanisms, heat conduction decreased with the raising amount of added water (ca. 30% for aerosol addition, ca. 10% for steam addition), convection was not significantly influenced, and the thermal radiation was reduced due to absorption by water molecules.

The resulting shares of heat transfer mechanisms, obtained by the difference method, were comparable to the literature values. Still, in order to obtain more reliable results in future work, the real emissivity of the test objects should be taken into account and the neglected heat transfer mechanisms should be more thoroughly analyzed. The proposed baking oven concept, tested in this work in a form of a model oven, has a relatively high thermal radiation component in comparison to a reference, state-of-art, electrically heated deck oven, which can be of advantage for reducing the baking time and increasing the oven's energy efficiency.

## Data Availability Statement

The datasets generated for this study are available on request to the corresponding author.

## Author Contributions

VJ developed the concept of novel baking oven with integrated VCBs, was leader of the research project, contributed to the experiment design and plan of the measurements and analysis, and discussion of obtained results. AZ-R proposed and created detailed research (based on the initial ideas of AD), administered and coordinated project activities, and contributed to the setup design, measurement plan, result analysis, discussion, review, and editing. BB developed the setup, conducted most of the measurements, and contributed to the characterization of the system and result analysis. AD provided the background idea for this research and significantly contributed to the research planning as a project coordinator and supervisor. All authors contributed to the article and approved the submitted version.

## Conflict of Interest

The authors declare that the research was conducted in the absence of any commercial or financial relationships that could be construed as a potential conflict of interest.

## Abbreviations

BCP: Black painted Cube on the baking Plate
BCI: Black painted Cube on the Insulation layer
C: heat transfer by convection
Cond: heat transfer by condensation
HC: heat transfer by heat conduction
NIR: near-infrared radiation range
PCP: Polished Cube on the baking Plate
PCI: Polished Cube on the Insulation layer
TR: heat transfer by thermal radiation
VCB: volumetric ceramic burner.
